# Chemotherapy with BCNU in recurrent glioma: Analysis of clinical outcome and side effects in chemotherapy-naïve patients

**DOI:** 10.1186/s12885-016-2131-6

**Published:** 2016-02-10

**Authors:** Christine Jungk, Despina Chatziaslanidou, Rezvan Ahmadi, David Capper, Justo Lorenzo Bermejo, Janina Exner, Andreas von Deimling, Christel Herold-Mende, Andreas Unterberg

**Affiliations:** Department of Neurosurgery, University Hospital Heidelberg, INF 400, 69120 Heidelberg, Germany; Institute of Neuropathology, University of Heidelberg, INF 224, 69120 Heidelberg, Germany; Institute of Medical Biometry & Informatics, University of Heidelberg, INF 305, 69120 Heidelberg, Germany

**Keywords:** Recurrent glioma, 1,3-bis (2-chloroethyl)-1-nitroso-urea (BCNU), Survival after relapse, Side effects, Pulmonary fibrosis

## Abstract

**Background:**

To date, standardized strategies for the treatment of recurrent glioma are lacking. Chemotherapy with the alkylating agent BCNU (1,3-bis (2-chloroethyl)-1-nitroso-urea) is a therapeutic option even though its efficacy and safety, particularly the risk of pulmonary fibrosis, remains controversial. To address these issues, we performed a retrospective analysis on clinical outcome and side effects of BCNU-based chemotherapy in recurrent glioma.

**Methods:**

Survival data of 34 mostly chemotherapy-naïve glioblastoma patients treated with BCNU at 1^st^ relapse were compared to 29 untreated control patients, employing a multiple Cox regression model which considered known prognostic factors including MGMT promoter hypermethylation. Additionally, medical records of 163 patients treated with BCNU for recurrent glioma WHO grade II to IV were retrospectively evaluated for BCNU-related side effects classified according to the National Cancer Institute Common Toxicity Criteria for Adverse Events (CTCAE) version 2.0.

**Results:**

In recurrent glioblastoma, multiple regression survival analysis revealed a significant benefit of BCNU-based chemotherapy on survival after relapse (*p* = 0.02; HR = 0.48; 95 % CI = 0.26–0.89) independent of known clinical and molecular prognostic factors. Exploratory analyses suggested that survival benefit was most pronounced in MGMT-hypermethylated, BCNU-treated patients. Moreover, BCNU was well tolerated by 46 % of the 163 patients analyzed for side effects; otherwise, predominantly mild side effects occurred (CTCAE I/II; 45 %). Severe side effects CTCAE III/IV were observed in 9 % of patients including severe hematotoxicity, thromboembolism, intracranial hemorrhage and injection site reaction requiring surgical intervention. One patient presented with a clinically apparent pulmonary fibrosis CTCAE IV requiring temporary mechanical ventilation.

**Conclusion:**

In this study, BCNU was rarely associated with severe side effects, particularly pulmonary toxicity, and, in case of recurrent glioblastoma, even conferred a favorable outcome. Therefore BCNU appears to be an appropriate alternative to other nitrosoureas although the efficacy against newer drugs needs further evaluation.

## Background

In newly diagnosed glioblastoma (GBM) World Health Organization (WHO) grade IV, maximum safe tumor resection followed by radio-chemotherapy with the alkylating agent temozolomide (TMZ) has been shown to be the most effective treatment and hence has evolved as standard therapy [1, 2]. At tumor recurrence, however, no standard of care has been defined so far. Therapeutic options have to be weighed carefully with regard to tumor size and location, clinical presentation and pre-treatment. Re-resection should be considered where appropriate; however, evidence of a favorable outcome is still poor due to heterogeneously pre-treated patients and many studies lacking standardized postoperative imaging [3–6]. Similarly, there are still a limited number of studies addressing re-irradiation for recurrent GBM [7–10].

Systemic chemotherapy is probably the most widely used salvage therapy for recurrent GBM though only a modest survival benefit has been demonstrated [11–17]. The interest in well-tolerated treatment regimens has grown due to a rising number of glioma patients pre-treated with TMZ, resulting in a reduced bone marrow reserve that may influence the efficacy and tolerance of additional chemotherapy. Nitrosourea derivatives, another class of alkylating agents, are widely applied in recurrent glioma even though their value remains controversial. In patients pre-treated with TMZ, there are few data available regarding the efficacy and tolerance of nitrosourea-based chemotherapy. Recent data demonstrated that the nitrosourea derivative ACNU alone failed to stabilize the disease in recurrent GBM [18] whereas ACNU in combination with teniposide (VM26) has been shown to be moderately effective in these patients but at the expense of an increased high-grade hematotoxicity [19]. For CCNU, another nitrosourea derivative, efficacy and safety was demonstrated both in newly diagnosed [20, 21] and recurrent [22] high-grade glioma. In North America the nitrosourea derivate BCNU (1,3-bis (2-chloroethyl)-1-nitroso-urea) historically has been applied more extensively both at initial diagnosis and at tumor recurrence than other nitrosourea derivatives. In Europe, BCNU lately experienced a renaissance after approval of ACNU has expired. As second-line chemotherapy, BCNU has been tested alone or in combination, among others with TMZ, irinotecan, cisplatin and thalidomide [23–27]. In a phase II trial conducted by Brandes et al. treating chemotherapy-naïve patients with recurrent GBM, BCNU-based chemotherapy was the only independent prognostic factor for a prolonged progression-free survival at 6 months (PFS-6) after onset of chemotherapy (17.5 %), however at the expense of long lasting hepatic and pulmonary toxicity [23]. In TMZ-pre-treated patients with recurrent GBM, BCNU in combination with irinotecan displayed a PFS-6 of 30.3 % with manageable toxicity [24]. In a recent retrospective analysis of 35 TMZ-pre-treated patients with recurrent GBM, a median PFS-6 of 13 %, a PFS of 11 weeks and an overall survival (OS) of 22 weeks after BCNU treatment were reported [28]. Common side effects of BCNU-based chemotherapy include nausea/vomiting and hematotoxicity with a delayed nadir after 4-6 weeks. The most dreaded side effect, however, is pulmonary fibrosis, leaving the preference of BCNU over other cytotoxic drugs controversial. Since data on BCNU-related side effects and its impact on patient outcome are still sparse and interpretation of study results is often hindered by a heterogeneously pre-treated patient sample, further evaluation of safety and efficacy in a large and homogeneously pre-treated cohort is warranted in order to consider BCNU as an appropriate treatment alternative.

To address these issues, we performed a retrospective analysis of 163 predominantly chemotherapy-naïve patients treated with BCNU for recurrent glioma WHO grade II to IV at the Department of Neurosurgery, University Hospital Heidelberg. Side effects were classified according to the National Cancer Institute Common Toxicity Criteria for Adverse Events (CTCAE) with special attention paid to pulmonary toxicity. In addition, clinical outcome was analyzed in 63 GBM patients with or without BCNU-based chemotherapy at tumor recurrence, adjusted for potential clinical (age, extent of resection (EOR) at 1^st^ surgery, TMZ at 1^st^ diagnosis, treatment intensity at tumor recurrence) and molecular MGMT (O^6^-methylguanine DNA methyltransferase) promoter hypermethylation) prognostic factors. Only IDH (isocitrate dehydrogenase) wildtype patients entered survival analysis, taking into account the unique molecular and prognostic phenotype associated with IDH mutations [29].

## Methods

### Patient sample

Medical records of glioma patients treated at the Department of Neurosurgery, University Hospital Heidelberg, were screened for demographic data (age, gender), Karnofsky Performance Scale score (KPS), histology, treatment regimens (e.g. surgery, radiotherapy, chemotherapy), and survival data. Information was collected in a Microsoft Access™ database. Written informed consent was obtained from each patient according to the research proposals approved by the Institutional Review Board at Heidelberg Medical Faculty.

#### Side effects

One hundred sixty-three patients were identified from this database having received BCNU-based chemotherapy for recurrent glioma WHO grade II to IV between 1995 and 2005. Medical records were screened for chemotherapy-related side effects that were classified according to the CTCAE version 2.0. Our in-house protocol included the intravenous administration of freshly prepared BCNU at 100 mg/m^2^ daily on two consecutive days every 6 - 8 weeks. Patients were followed by blood tests every other week as well as chest X-rays for the development of pulmonary fibrosis and MRI scans for tumor response every 3 months. Dose reduction was performed at the physician’s discretion when patients presented with severe hematotoxicity, renal dysfunction or a poor physical condition. Efficacy of BCNU-based chemotherapy was determined for each WHO grade employing the Kaplan-Meier method. Progression-free survival after BCNU (PFS_BCNU_) was defined as the time interval between onset of BCNU treatment and change of treatment or death, whatever occurred first, and overall survival (OS) was defined as the time interval between histological diagnosis and death. Subjects were censored to survival analysis if the corresponding event (PFS_BCNU_: change of treatment after BCNU-based chemotherapy/death; OS: death) was not observed during follow-up (until April 2015).

#### Outcome

For in-depth univariate and multiple survival analyses addressing the efficacy of BCNU-based chemotherapy in recurrent GBM, 135 patients with recurrent GBM treated at our institution between 1995 and 2005 were identified from our database. 72 patients were excluded due to insufficient documentation, missing follow-up information, presence of IDH mutation or lack of tissue samples to determine MGMT promoter methylation status. The remaining 34 cases with (BCNU group) and 29 cases without (control group) BCNU treatment at 1^st^ tumor relapse entered survival analyses. A patient was considered to have recurrent disease if this was revealed either by MRI or neurological deterioration, leading to an adaption of anti-tumor therapy. Hence, PFS was defined as the time interval between histological diagnosis and tumor recurrence and survival after relapse as the time interval between tumor recurrence and death. All patients died during follow up. Estimated hazard ratios were adjusted for established prognostic factors (patient’s age at diagnosis, KPS at tumor recurrence, EOR at 1^st^ surgery, TMZ at 1^st^ diagnosis) and other potential confounders (therapies other than BCNU at tumor recurrence). In all cases, the EOR was determined by MRI scans taken within 72 h after surgery, and complete resection (CR) was defined as no residual contrast-enhancing tumor. Furthermore, MGMT promoter hypermethylation, a molecular marker predictive of the treatment response to alkylating agents like nitrosoureas and temozolomide [30, 31], was also included in the multiple regression analysis.

### Molecular markers

IDH1 R132H mutation was ruled out by immunohistochemistry as previously described [32]. Due to the reported low frequency of IDH mutations in primary glioblastomas, cases negative for IDH1 R132H immunohistochemistry were designated as IDH wildtype. For confirmation, we performed direct sequencing of the mutation hotspot regions of IDH1 (*n* = 23) and IDH2 (*n* = 10) for selected cases as described [33]. As expected, no rare IDH1 or IDH2 mutations were detected by sequencing among these cases. MGMT promoter methylation status was determined by methylation-specific polymerase chain reaction as previously described [34].

### Statistical analysis

Statistical evaluation of BCNU-related side effects was performed employing Microsoft Excel™ software. Univariate survival analysis was based on the Kaplan-Meier method and multiple survival analyses relied on proportional hazard regression models, where BCNU and other therapies after relapse were treated as time-dependent variables. Statistical analyses were conducted using SAS version 9.2 and Kaplan-Meier curves were plotted using R version 2.11.1 (The R Project for Statistical Computing, http://www.r-project.org/). Group differences were assessed with the nonparametric Mann–Whitney test for continuous variables and with Fisher’s exact and Chi-square tests for ordinal scaled variables using Graph-Pad Prism software (Version 5.0c, Graph Pad Inc., CA, USA). *P*-values ≤ 0.05 were considered statistically significant.

## Results

### Analysis of BCNU-related side effects

No standard of care exists for recurrent glioma, but nitrosourea derivatives, among others BCNU, are considered appropriate options. We identified 163 patients from our database treated with BCNU for recurrent glioma WHO grade II to IV and retrospectively evaluated their medical histories in view of possible BCNU-related side effects. Median age at the onset of BNU-based chemotherapy was 44 years (range 17–81 years) with a male preponderance of 2:1. Median KPS (defined as lowest KPS observed during the treatment period) was 80 %. Apart from BCNU, 147 patients (90 %) received irradiation and 20 (12.2 %), 4 (2.4 %) and 2 (1.2 %) patients TMZ, PCV (procarbazine, CCNU, vincristine) and methotrexate, respectively (Table 1). BCNU was administered in a median total dose of 1662 mg (range 300–5200 mg) during a median number of 5 cycles (range 1–16 cycles). Dose reduction was necessary in 48 patients (29.4 %) due to hematotoxicity, renal dysfunction or a poor physical condition (Table 1). Most patients received BCNU for recurrent high-grade glioma WHO grade IV (51.5 %) and III (35 %), in the latter preferably with an oligodendroglial component (oligodendroglioma (18.4 %) and mixed glioma (8.6 %) WHO grade III) (Table 1).

**Table 1:** Baseline characteristics and efficacy data of patient cohort analyzed for BCNU-related side effects

Patient Characteristics	
Patient Sample [n]	163
Age at onset of BCNU therapy [years] (median; range)	44 (17–81)
Sex [male:female; n]	108:55
Karnofsky Performance Score [%] (median; range)	80 (20–100)
Histology [number of patients] (%)	
Glioblastoma WHO grade IV	84 (51,5)
Oligodendroglioma WHO grade III	30 (18,4)
Oligoastrocytoma WHO grade III	14 (8,6)
Astrocytoma WHO grade III	13 (8)
Oligodendroglioma WHO grade II	12 (7,4)
Oligoastrocytoma WHO grade II	3 (1,8)
Astrocytoma WHO grade II	7 (4,3)
Median OS [months]	
WHO grade II tumors	191
WHO grade III tumors	144
WHO grade IV tumors	20
Median PFS_BCNU_ [months]	
WHO grade II tumors	85
WHO grade III tumors	28
WHO grade IV tumors	7
Death [number of patients] (%)	
WHO grade II tumors	9 (41)
WHO grade III tumors	37 (65)
WHO grade IV tumors	82 (98)
Lost to follow up [number of patients] (%)	
WHO grade II tumors	5 (23)
WHO grade III tumors	13 (23)
WHO grade IV tumors	1 (1)
Pre-treatment [number of patients] (%)	
Radiotherapy	147 (90)
Temozolomide	20 (12,2)
PCV	4 (2,4)
Methotrexate	2 (1,2)
BCNU – total dose [mg] (median; range)	1662 (300–5200)
BCNU – number of cycles (median; range)	5 (1–16)
BCNU-related side effects [number of patients] (%)	88 (54)
WHO grade II tumors	18 (82)
WHO grade III tumors	35 (61)
WHO grade IV tumors	35 (42)
BCNU – dose reduction to due side effects (%)	48 (29,4)
BCNU – Hospital admission due to side effects (%)	2 (1,2)
Chemotherapy-related deaths (%)	0

**Fig. 1:**
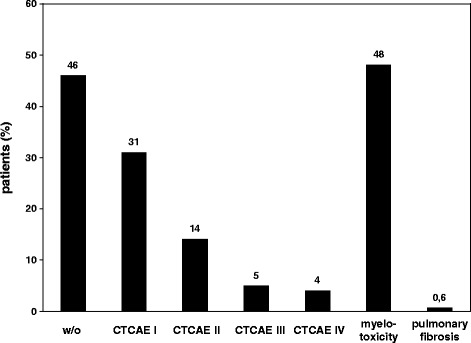
BCNU-related side effects as observed in 163 patients treated for recurrent glioma WHO II – IV. Side effects were classified according to the CTCAE version 2.0 and are plotted on the x-axis

Eighty-eight of 163 patients (54 %) experienced BCNU-related side effects (Fig. 1). The frequency of side effects was not equally distributed among tumor grades, with WHO grade IV patients experiencing least (42 %) and WHO grade II patients experiencing most frequently (82 %) side effects (Table 1). In general, BCNU was well tolerated since mainly mild side effects CTCAE I/II (45 % of all patients) occurred, predominantly due to myelosuppression (48 % of all patients with side effects; Fig. 1) resulting in leucopenia (33 %), thrombocytopenia (24 %) or anemia (9.6 %). Otherwise CTCAE I/II side effects consisted of nausea/vomiting, fatigue, obstipation/diarrhea and injection site reaction. Severe side effects CTCAE III/IV were rarely observed (9 % of all patients) including myelosuppression (6 %), thromboembolism (one patient with a deep venous thrombosis and pulmonary embolism each; 1.2 %), intracranial hemorrhage due to a chronic subdural hematoma (0.6 %), and injection site reaction requiring surgical intervention (0.6 %) (Table 2). One out of 163 patients (0.6 %) presented with a clinically apparent pulmonary fibrosis CTCAE IV requiring temporary mechanical ventilation, otherwise routine chest X-rays and clinical examination revealed no signs of pulmonary fibrosis (Fig. 1). Side effects classified as “others” were reported in timely correlation to the administration of BCNU but were not explicitly related to it (Table 2). Hospital admission due to BCNU-related side effects was necessary in 2 patients (1.2 %) (Table 1). There were no BCNU-related deaths (Table 1).

**Table 2:** Side effects of BCNU-based chemotherapy classified according to CTCAE v.2.0

Side Effects (%)	CTCAE I	CTCAE II	CTCAE III	CTCAE IV
Patients with side effects	51 (31)	23 (14)	8 (5)	7 (4)
Leukopenia	38 (22,8)	17 (10,2)	2 (1,2)	1 (0,6)
Thrombocytopenia	35 (21)	5 (3)	5 (3)	1 (0,6)
Anemia	16 (9,6)	-	1 (0,6)	-
Nausea/Vomitus	3 (1,8)	2 (1,2)	-	-
Fatigue	1 (0,6)	-	-	-
Obstipation/Diarrhea	2 (1,2)	-	-	-
Pulmonary Fibrosis	-	-	-	1 (0,6)
Thromboembolism	-	-	1 (0,6)	1 (0,6)
Hemorrhage	-	-	-	1 (0,6)
Injection Site Reaction	1 (0,6)	2 (1,2)	1 (0,6)	-
Others^a^	4 (2,4)	2 (1,2)	2 (1,2)	1 (0,6)

^a^Side effects classified as “others” were found in timely but not necessarily causal relation to BCNU administration and consisted of weight loss/loss of appetite CTCAE I (1.2 %), arterial hypotension CTCAE I (0.6 %), neuropathic pain CTCAE I (0.6 %), newly observed cranial nerve deficit CTCAE II (0.6 %), photophobia CTCAE II (0.6 %) and hallucinations CTCAE III (0.6 %), anaphylaxis CTCAE III (0.6 %) and isolated elevation of liver transaminases CTCAE IV (0.6 %)

### Favorable outcome of patients treated with BCNU for recurrent glioblastoma

Efficacy data of all patients analyzed for BCNU-related side effects are listed in Table 1. PFS_BCNU_ was 85, 28 and 7 months for WHO grade II, III and IV gliomas, respectively. However, clinical courses and treatment plans were very heterogeneous and outcome-related molecular markers were available for a minority of patients only. We therefore decided to focus on the impact of BCNU-based chemotherapy on patient outcome in recurrent GBM and analyzed a well-defined, homogeneously pre-treated, mostly chemotherapy-naïve sample of 63 patients both by univariate and multiple survival regression models. At initial diagnosis, all patients were pre-treated with maximum safe tumor resection followed by irradiation. At 1^st^ relapse, patients underwent surgery where appropriate followed by administration of BCNU (BCNU group; *n* = 34 patients) or not (control group; *n* = 29 patients). All but 5 patients in each group were chemotherapy-naïve by the time of tumor recurrence; these patients received TMZ at initial diagnosis. Salvage therapies at relapse apart from BCNU included (BCNU group/control group; n): re-resection (9/1), re-irradiation (4/5), TMZ (4/1), thalidomide (1/0) and CCNU (1/0). Thus, treatment intensity at relapse was higher in the BCNU group than in the control group (38.2 % versus 24.1 %; *p* = 0.28, Fisher’s exact test) and therefore was included as a potential time-dependent confounder in multiple survival analysis. Since re-irradiation and chemotherapy were not necessarily considered therapeutic options for recurrent GBM during the time our patients were treated and particularly TMZ was not available outside clinical trials, BCNU was offered based on individual decisions with risks and benefits carefully weighed against each other, often at the patient’s request. 88.2 % of patients received ≤ 6 cycles of BCNU, 2 patients received more than 6 cycles and in 2 patients the exact cycle number was not reliably determinable. Median age was 56 years for BCNU patients and 62 years for control patients (*p* = 0.06; Mann–Whitney test). Median KPS at tumor recurrence was 80 % in the BCNU group and 60 % in the control group based on patients with available KPS information; however, due to the retrospective study design reliable KPS information could not be determined for all patients and was not considered in multiple regression analyses. Frequency of CR at 1^st^ diagnosis was similar in both groups (BCNU: 32 %; control: 31 %); otherwise, surgical procedures consisted of biopsy or subtotal resection (Table 3).

**Table 3:** Description of patient cohort included in survival analysis (*n* = 63 patients)

	BCNU	Control
Patient sample (n)	34	29
Age at 1^st^ diagnosis (median; range)	56 (22–76)	62 (33–78)
Sex (male: female; n)	20:14	16:13
KPS (median; range)	8 (4–10)	6 (3–9)
MGMT promoter hypermethylation (%)	64,7	55,2
Overall survival (median: days (months))	480 (15)	429 (14)
Progression-free survival (median: days (months))	186 (6)	180 (5)
Survival after relapse (median: days (months))	266 (9)	187 (6)
Complete resection at 1^st^ surgery (%)	32,4	31
Radiotherapy at 1^st^ diagnosis (%)	100	100
TMZ at 1^st^ diagnosis (%)	14,7	17,2
Therapies other than BCNU at recurrence (re-resection, re-irradiation, TMZ, CCNU, thalidomide)	38,2	24,1
BCNU cycles (% of patients)		N/A
- ≤6	88,2	
- 7–10	5,9	
- not determinable	5,9	

N/A: not applicable

**Fig. 2:**
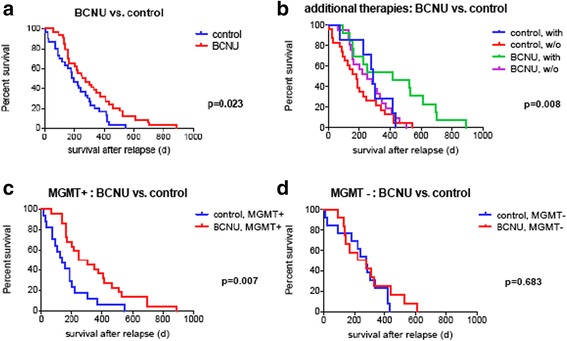
Kaplan-Meier plots depicting survival after relapse of 63 patients treated with (“BCNU”) or without (“control”) BCNU after recurrent GBM. Note that the association of BCNU treatment with an improved survival after relapse (a) was even more pronounced in patients with other therapies than BCNU at tumor recurrence (“with” compared to “w/o” (without); b) as well as in patients with hypermethylated MGMT promoter (c) while patients with unmethylated MGMT promoter did not seem to benefit from BCNU treatment (d)

PFS was comparable in both groups (BCNU: median 186 days; control group: median 180 days; *p* = 0.78, Log-rank test). However, BCNU-based chemotherapy conferred a significant impact on survival after relapse (BCNU: median 266 days; control group: median 187 days; *p* = 0.02, Log-rank test; Fig. 2a). In order to take into account clinical (age at 1^st^ diagnosis, EOR at 1^st^ surgery, TMZ at 1^st^ diagnosis, therapies other than BCNU at tumor recurrence) and molecular (MGMT promoter methylation) prognostic factors as potential confounders, a multiple Cox proportional hazard analysis was performed. Results confirmed BCNU treatment as independent prognostic factor for prolonged survival after relapse (*p* = 0.02; HR = 0.48; 95 % CI = 0.26–0.89; Table 4). Age at 1^st^ diagnosis (*p* = 0.04; HR = 1.03; 95 % CI = 1.00–1.05; Table 4) and TMZ at 1^st^ diagnosis (*p* = 0.005; HR = 0.32; 95 % CI = 0.15–50.7; Table 4) simultaneously showed a significant impact on survival after relapse. We also found some independent evidence on a prolonged survival after relapse of patients treated with other therapies than BCNU (*p* = 0.06; HR = 0.56; 95 % CI = 0.30–1.02). In univariate exploratory analyses, survival after relapse differed in BCNU and control patients with and without (w/o) additional therapies at relapse (BCNU | with: 413 days; control | with: 290 days; BCNU | w/o: 251 days; control | w/o: 181 days; Fig. 2b).

**Table 4:** Prognostic factors of survival after tumor relapse (“survival after relapse”) based on a multiple Cox regression model (*n* = 63 patients)

Variable		Hazard ratio^a^	95 % CI	*p*-value
Age at 1^st^ diagnosis	per year	1.03	1.00–1.05	0.04
EOR	CR vs. PR	0.76	0.41–1.40	0.64
TMZ at 1^st^ diagnosis	yes vs. no	0.32	0.15–0.71	0.005
Therapies other than BCNU at tumor recurrence	yes vs. no	0.56	0.30–1.02	0.06
MGMT promoter methylation	yes vs. no	0.89	0.51–1.53	0.66
BCNU	yes vs. no	0.48	0.26–0.89	0.02

^a^A hazard ratio <1 (>1) indicates an effect in favor of the first (second) group

*CI* confidence interval, *EOR* extent of resection, *CR* complete resection, *PR* partial resection/biopsy

Notably, exploratory analysis revealed that the effect of BCNU on survival after relapse was most pronounced for MGMT-hypermethylated patients (*n* = 38; *p* = 0.007; Fig. 2c). In patients with non-methylated MGMT promoters (*n* = 25), no significant difference in survival after relapse by BCNU treatment was observed (*p* = 0.68; Fig. 2d).

## Discussion

In this study, chemotherapy with the nitrosourea derivate BCNU for the treatment of recurrent glioma was both effective and well tolerated. In a homogeneously pre-treated, mostly chemotherapy-naïve sample of 63 patients suffering from recurrent IDH wildtype GBM, outcome analysis revealed a survival benefit for patients treated with BCNU at 1^st^ tumor relapse since survival after relapse was significantly prolonged compared to control patients (BCNU: 266 days; control: 187 days; *p* = 0.02). This survival benefit was most pronounced for MGMT-hypermethylated, BCNU-treated patients (*p* = 0.007). Even though median age, a known prognostic factor for a favorable outcome [35, 36], was lower (56 years versus 62 years; *p* = 0.06) and treatment intensity at tumor recurrence was higher (38 % versus 24 % of patients receiving salvage therapies; *p* = 0.28) in the BCNU group, multiple regression suggested BCNU-based chemotherapy to be an independent prognostic factor of prolonged survival after relapse (HR = 0.48; p = 0.02).

These data are supported by a phase II trial conducted by Brandes et al. [23] that treated 40 patients suffering from recurrent GBM with BCNU given for up to 6 cycles alone or in combination with re-resection. PFS-6 was 17.5 % and median OS from the onset of chemotherapy was 7.53 months (equivalent to the terminus “survival after relapse” in our study). Response to chemotherapy was the only independent prognostic factor for PFS-6, whereas KPS and previous histology of low-grade glioma were the only independent prognostic factors for OS. Our survival data are also comparable to those of other phase II trials for recurrent GBM analyzing the effect of BCNU in combination with other drugs (Table 5). In a retrospective analysis of 35 mostly TMZ-pre-treated patients with recurrent GBM, Reithmeier et al. reported slightly inferior survival data with PFS-6 of 13 %, PFS of 11 weeks and OS of 22 weeks after BCNU treatment [28]. Noteworthy, these patients received a mean of 1.8 cycles BCNU only, pre-treatment at tumor relapse was very heterogeneous and commencement of BCNU therapy varied between the 1^st^ and 4^th^ relapse. Interestingly, in a multivariate analysis no influence of TMZ pre-treatment on patient outcome was found, tempting the authors to question the concern that pre-treatment with another alkylating agent such as TMZ might not only increase toxicity but also reduce the efficacy of nitrosoureas due to an acquired drug resistance. However, like in the majority of studies, the significance of this finding is impaired by the lack of the MGMT promoter methylation status. In a meta-analysis of 504 cohorts with 24 193 patients Wolff and co-workers reported ACNU- and CCNU-containing regimens to be superior to BCNU in terms of OS, even though the different nitrosourea-treated cohorts were not comparable due to variations in treatment regimens and histology [15]. BCNU was predominantly applied as monotherapy for recurrent GBM whereas ACNU and CCNU were administered in combination with other drugs in newly diagnosed high-grade gliomas [15]. Beside, in a single center study of TMZ-pretreated patients with recurrent GBM, ACNU alone or in combination with other drugs failed to induce a significant stabilization of disease, however at the expense of 50 % high-grade hematotoxicity [18]. Hence a potential advantage of one nitrosourea derivate over another still needs to be determined, particularly in a homogeneously pre-treated patient sample. It has to be noted, however, that recent meta-analyses of predominantly phase II trials for the use of TMZ or the antiangiogenic agent bevacizumab in patients with recurrent GBM reported PFS-6 rates superior to those of the previously discussed nitrosourea studies [37, 38].

**Table 5:** Synopsis of selected phase II chemotherapy trials performed for recurrent GBM

Reference	Intervention	Pts. (n)	chemo-naïve (%)	PFS_6 mo_ (%)	PFS (weeks)
Brandes (2004) [23]	BCNU ± re-resection	40	100	17.5	13.3
Fine (2003) [25]	BCNU + thalidomide	38	50	27	14.9
Prados (2004) [26]	BCNU + TMZ (single dose)	38	89.5	21	11
Brandes (2004) [24]	BCNU + irinotecan	42	0	30.3	17
Yung (2000) [46]	TMZ (5/28) vs. procarbazine	112	35	21 vs. 8	12.4
Brada (2001) [47]	TMZ (5/28)	128	29	18	8
Brandes (2002) [48]	TMZ (5/28)	42	0	24	11.7
Chang (2004) [49]	TMZ (5/28)	142	56	18	10
Wick (2007) [50]	TMZ (7/14)	64	36	43.8	24
Perry (2010) [51]	TMZ (continuous)	91	NR	23.9	7–15
Kappelle (2001) [52]	PCV	63	68.2	29	13
Wong (1999) [53]	historical controls	225	NR	15	9

*ND* not determined, *NR* not reported

The observational, retrospective design of our survival analysis confers some disadvantages, including a potential selection bias by comparing two groups of patients compiled on the basis of availability of outcome data and tumor tissue, uncontrolled for known and suspected prognostic factors. Accordingly, median age was non-significantly lower and treatment intensity at tumor relapse was non-significantly higher in the BCNU group, but we used multiple regression analysis to take into account this imbalance. Although the sample size was relatively small to simultaneously assess six prognostic factors, BCNU treatment consistently proved to be an independent prognostic factor for a prolonged survival after relapse. The present study also has some strengths. We analyzed a homogeneously pre-treated, mostly chemotherapy-naïve cohort of patients with uniform histology (recurrent GBM). Importantly, only patients with IDH wildtype GBM were included, eliminating the unique molecular and prognostic phenotype variability related to IDH mutant GBM [29]. In contrast to other studies, MGMT promoter methylation status, which is predictive for the response of GBM to alkylating drugs like TMZ and nitrosoureas [31, 39, 40], was included in the multivariate model, showing an even pronounced survival benefit for MGMT-hypermethylated, BCNU-treated patients. Moreover, the EOR at 1^st^ surgery was objectively quantified by routine post-operative MRI scans and was included in the multivariate model as well. This is of importance since complete resection of newly diagnosed GBM has been shown to be a positive prognostic factor for a prolonged survival [2, 41–44].

Given the poor prognosis of high-grade gliomas, especially after tumor relapse, quality of life experienced by these patients is an important issue. However, many second-line chemotherapy regimens are highly toxic. In our analysis of chemotherapy-related side effects, BCNU was well tolerated. Interestingly, side effects were not equally observed among tumor grades, with WHO grade IV patients experiencing least and WHO grade II patients experiencing most frequently side effects. It is worth noting that treatment intensity (radiotherapy, chemotherapy) prior to BCNU-based chemotherapy increased with WHO grade (data not shown). The retrospective nature of our study does not allow an explanation, but this finding may be due to the considerably extended life span of WHO grade II patients compared to higher-grade glioma patients with an increasing likelihood of observing side effects. Out of 163 patients treated with BCNU for recurrent glioma WHO grade II to IV, 54 % experienced mostly mild chemotherapy-related side effects, predominantly due to myelosuppression. Severe side effects CTCAE III/IV were observed in 9 % of all patients including hematotoxicity, thromboembolism, intracranial hemorrhage and injection site reaction requiring surgical intervention. Only one patient presented with the most dreaded side effect of BCNU administration, a clinically apparent pulmonary fibrosis CTCAE IV requiring temporary mechanical ventilation. These data are comparable or even superior to other multimodal treatment regimens in chemotherapy-naïve patients with newly diagnosed GBM (Table 6). The EORTC 22981/26981 trial [1] reported hematotoxicity CTCAE III/IV in 16 % of patients undergoing postoperative radio-chemotherapy with TMZ. As for nitrosourea-based regimens, Buckner et al. observed leucopenia and thrombocytopenia CTCAE III/IV in 28 % and 44 % of patients treated with BCNU ± radiotherapy [27], and the NOA-01 trial reported hematotoxicity CTCAE III/IV in 36.5 % of patients exposed to ACNU/VM 26 in combination with radiotherapy [45]. In contrast, we observed severe leucopenia and thrombocytopenia CTCAE III/IV in 1.8 and 3.6 % of patients only. These data are in line with the phase II trial by Brandes et al. in which WHO grade 3/4 leucopenia and thrombocytopenia were observed in 8 % and 10 % of cycles, respectively [23]. However, the authors reported a high incidence of pulmonary (WHO grade 4: 5 % of patients) and hepatic (WHO grade 2/3: 10 % of patients) toxicity, leading them to the conclusion that even though patient outcome was comparable to similar phase II trials with TMZ as single agent, BCNU-associated toxicity was more frequent and persistent. This study monitored pulmonary function by diffusing capacity of the lung for carbon monoxide. In contrast, the NCCTG/SOG trial conducted by Buckner et al. made use of clinical examination and chest X-rays every other month and reported adverse pulmonary events not further specified in 10 % of patients [27]. Finally, in the NOA-01 trial pulmonary function was monitored merely by clinical examination and patient’s history, detecting pulmonary fibrosis in 0.7 % of patients [45]. Thus, studies analyzing pulmonary toxicity of nitrosourea-based chemotherapy, especially with a focus on pulmonary fibrosis, are difficult to compare due to heterogeneous monitoring techniques of pulmonary function. In our analysis of side effects, we screened medical records of a large, predominantly chemotherapy-naïve patient sample for reports on clinical symptoms of pulmonary dysfunction; furthermore, routine chest X-rays every 3 months were part of our in-house protocol for patients exposed to BCNU-based chemotherapy. Therefore, even though our study design was a retrospective one, pulmonary monitoring is comparable to the one employed by Buckner et al. [27]. However, the incidence of pulmonary fibrosis detected both clinically and radiographically was much lower (0.6 %; 1 out of 163 patients) and is actually in the range of ACNU-related pulmonary toxicity reported by Weller et al. [45]. Our data suggest that a clinically apparent pulmonary fibrosis caused by BCNU might be less frequent than previously feared and that BCNU, in this respect, is not inferior to other nitrosourea derivatives. Nevertheless, a more sensitive monitoring instrument of pulmonary function would be desirable in order to strengthen this notion.

**Table 6:** BCNU-related side effects - Comparison of own results to the literature

Reference	Cytotoxic agent	Hematotoxicity	Pulmonary SE	CTH-related deaths (%)
		CTCAE III/IV (%)	CTCAE I - IV (%)	
Stupp (2005) [1]	TMZ (+RT)	16	-	-
Buckner (2006) [27]	BCNU (+ RT)	L 28	10	-
		T 44		
Weller (2003) [45]	BCNU (+WBRT)	-	12	4
Weller (2003) [45]	ACNU/VM26 (+RT)	36,5	0,7	2,6
Brandes (2004) [23]	BCNU	L 8		ND
		T 10	5	
		(WHO 3/4)	(WHO 4)	
own results	BCNU	L 1,8	0,6	0
		T 3,6		

*SE* side effects, *CTCAE* Common Toxicity Criteria, *CTH* chemotherapy, *L* leukopenia, *T* thrombocytopenia, *RT* radiotherapy, *WBRT* whole brain radiotherapy

## Conclusions

In our analysis of BCNU-based chemotherapy in patients with recurrent glioma, BCNU was well tolerated and, in case of recurrent GBM, even conferred a significant survival benefit. If these encouraging results hold true in nowadays TMZ-pre-treated patients, still needs to be determined. Since no standard therapy exists for recurrent high-grade glioma and a survival benefit of other nitrosoureas over BCNU has not been proven yet, we propose to further evaluate its efficacy in future prospective trials.

## Abbreviations

BCNU: 1,3-bis (2-chloroethyl)-1-nitroso-urea
CTCAE: Common Toxicity Criteria for Adverse Events
HR: Hazard Ratio
CI: Confidence Interval
GBM: Glioblastoma
WHO: World Health Organization
TMZ: Temozolomide
VM26: Teniposide
PFS-6: Progression - Free Survival at 6 Months
KPS: Karnofsky Performance Score
OS: Overall Survival
PFS: Progression - Free Survival
EOR: Extent of Resection
IDH1: Isocitrate Dehydrogenase 1
MGMT: O^6^-Methylguanine DNA Methyltransferase
PCV: Procarbazine/CCNU/Vincristine
EORTC: European Organisation for Research and Treatment of Cancer
NCCTG/SOG: North Central Cancer Treatment Group/Southwest Oncology Group.
